# A DNA Prime-Inactivated Boost Regimen Enhances Immunogenicity Against Pigeon Newcastle Disease: A Comparative Study and Analysis of Synergistic Effects

**DOI:** 10.3390/vetsci13030251

**Published:** 2026-03-09

**Authors:** Shuai Luo, Yiyi Ren, Nikolai Vladimirovich Tarlavin, Dmitrii Andreevich Kraskov, Edward Javadovich Javadov, Da Xu, Houqiang Luo, Suzhen Liu

**Affiliations:** 1 College of Animal Sciences, Wenzhou Vocational College of Science and Technology, Wenzhou 325000, China; luoshuai@wzvcst.edu.cn (S.L.); renyiyi@wzvcst.edu.cn (Y.R.); xd13666557571@outlook.com (D.X.); 2 All-Russian Scientific Research Institute of Poultry Processing Industry—Branch of the Federal Scientific Center ‘All-Russian Research and Technological Poultry Institute’ of the Russian Academy of Sciences, Saint Petersburg State University of Veterinary Medicine, St. Petersburg 190000, Russia; tarlav1995@bk.ru (N.V.T.); kraskov-00@bk.ru (D.A.K.); vnivip1@mail.ru (E.J.J.)

**Keywords:** pigeon Newcastle disease, DNA vaccine, inactivated vaccine, synergistic strategy, immunization

## Abstract

Pigeon Newcastle disease poses a persistent threat to the global pigeon industry, underscoring the need for effective vaccination strategies. While both inactivated and DNA vaccines offer distinct advantages, the immunogenicity of a combined heterologous regimen remains underexplored. This study evaluated and compared three immunization strategies in pigeons: a DNA vaccine encoding the NDV F protein fused with chicken IL-18, an inactivated vaccine from a local virulent strain, and a DNA prime-inactivated boost regimen. The preparation workflows for both vaccine platforms are described in detail to provide methodological context for the immunological comparison. Critically, the prime–boost regimen elicited significantly higher hemagglutination inhibition (HI) antibody titers than either vaccine administered alone, demonstrating a clear synergistic effect. These findings highlight the complementary roles of the two platforms and provide a strong immunological rationale for further evaluation of this sequential strategy. Future studies incorporating viral challenge experiments and long-term immune monitoring are needed to determine whether the enhanced HI antibody response translates into protective efficacy under field conditions.

## Simple Summary

This study compares the immunogenicity of two vaccine platforms—DNA vaccine and inactivated vaccine—when used alone or in a prime–boost regimen for protecting pigeons against Newcastle disease. The DNA vaccine was engineered to express the viral fusion protein fused to chicken IL-18 as an immunostimulant, whereas the inactivated vaccine was prepared from a local virulent strain. Detailed preparation protocols for both vaccines are provided. Researchers tested four groups of pigeons: one received the DNA vaccine, one received the inactivated vaccine, one received both in a prime–boost strategy (DNA first, then inactivated), and one was a control group. Results showed that both vaccines alone induced good immune responses, but the combination of DNA prime and inactivated boost produced significantly higher antibody levels than either vaccine alone. The study emphasizes the combined strengths of both platforms and may provide a basis for future studies to improve vaccination strategies against pigeon Newcastle disease.

## 1. Introduction

Pigeon Newcastle disease, a highly contagious viral avian disease, continues to threaten the global pigeon industry [1,2]. Effective control is crucial, impacting animal welfare, economic sustainability, food security, and public health. Vaccination stands as the primary preventive measure, with inactivated vaccines and DNA vaccines representing two leading technological platforms, each featuring pronounced strengths and limitations [3,4,5]. Inactivated vaccines are valued for their strong immunogenicity and technological maturity, yet they typically depend on adjuvants to boost immunity and involve the risks associated with live-virus handling during manufacturing [6]. Conversely, DNA vaccines offer advantages in design flexibility, rapid production, and enhanced safety profiles [7], though their translational success is often hampered by challenges in delivery efficiency and immunogenic potency.

Previous studies in avian vaccinology have explored heterologous prime–boost strategies using various platform combinations. For instance, DNA prime followed by inactivated vaccine boost has been shown to enhance immune responses against infectious bronchitis virus (IBV) in chickens [8], and similar approaches have been investigated for Newcastle disease virus using different vector combinations [9,10]. However, the preparation workflows for inactivated and DNA vaccines have been individually described; thus, a direct comparison of their immunogenicity—particularly in the context of a heterologous prime–boost strategy—remains underexplored. In this study, we developed both a DNA vaccine (encoding the NDV F protein fused with chicken IL-18) and an inactivated vaccine (based on a local pigeon NDV isolate), with detailed protocols provided for each platform. Using these well-characterized vaccines, we then conducted an in vivo immunogenicity study in pigeons to compare three immunization strategies: DNA vaccine alone, inactivated vaccine alone, and a DNA prime-inactivated boost regimen. The primary endpoint was NDV-specific hemagglutination inhibition (HI) antibody titers, a key correlate of protective immunity. We evaluate their complementary potential across stages and discuss possibilities for combined or sequential immunization strategies. By presenting this integrated overview, the work aims to serve as a practical reference for advancing the next generation of avian disease vaccines.

However, these studies were conducted exclusively in chickens using chicken-adapted NDV strains. Given the host-adapted nature of pigeon paramyxovirus type 1 (PPMV-1) and its antigenic distinctiveness from classic NDV strains, the immunogenicity and potential synergy of a DNA prime-inactivated boost regimen in pigeons remain unexplored. This study, therefore, aimed to fill this gap by evaluating this heterologous strategy in pigeons using a genotype VII PPMV-1 isolate.

## 2. Materials and Methods

### 2.1. Experimental Materials and Reagents

The following materials and reagents were utilized in this study: standard laboratory consumables, including 1.5 mL microcentrifuge tubes, 5 mL and 10 mL centrifuge tubes, pipette tips (10 μL, 200 μL, 1 mL), 0.22 μm syringe filters (domestic), a vortex mixer, 1 mL disposable syringes, No. 3 disposable injection needles, 75% ethanol, cotton balls, masks, and gloves. Materials for animal housing and handling included empty cages, feed, and water. A fresh 1% chicken red blood cell suspension was prepared in-house. Newcastle disease virus (NDV)-positive serum was commercially obtained from Guosheng Biotechnology Co., Ltd. (Harbin, China). The pigeons used in this study (Loman White breed, sourced from a local commercial farm) are described in detail in Section 2.5.

### 2.2. DNA Vaccine: Reagents and Materials

Key enzymes and kits for molecular cloning were obtained from commercial suppliers: T4 DNA ligase and restriction endonucleases (*Bam*HI and *EcoR*I) were purchased from New England Biolabs (Ipswich, MA, USA). Plasmid DNA extraction and purification were performed using the QIAprep Spin Miniprep Kit and the EndoFree Plasmid Mega Kit (QIAGEN, Hilden, Germany). Competent E. coli DH5α cells were acquired from Weidi Biotechnology (Shanghai, China). All other general biochemical reagents were of analytical grade.

### 2.3. DNA Vaccine Preparation Protocol

#### 2.3.1. Antigen Gene Selection and Plasmid Vector Construction

The target antigen gene was selected based on its critical role in eliciting protective immunity against avian paramyxovirus 1 (pigeon strain). The gene encoding the major protective antigen, the fusion (*F*) protein (GenBank accession no. MK516201.1), was chosen for vaccine construction. To potentially enhance cellular immune responses, a segment of *Gallus IL-18* (GenBank accession no. AY775780.1) was incorporated into the design, creating a fusion gene construct.

#### 2.3.2. Plasmid Vector and Synthesis

The eukaryotic expression vector pCMV (containing a CMV promoter and an ampicillin resistance cassette) was used as the backbone. The codon usage of the selected viral antigen gene(s) was optimized for high expression in avian or mammalian cells using dedicated algorithms (GenScript (Piscataway, NJ, USA)). Undesired cis-acting motifs (such as cryptic splice sites or instability sequences) were removed. The optimized gene sequence was synthesized de novo with flanking restriction enzyme sites (*Bam*HI and *EcoR*I) for directional cloning. The recombinant plasmid, designated pCMV-*IL-18*/NDV-*F*, encodes the NDV F protein fused to chicken IL-18 under the control of the CMV promoter.

#### 2.3.3. Cloning and Verification

The synthesized gene fragment and the plasmid vector were digested with the corresponding restriction enzymes. The digested products were purified, ligated using T4 DNA ligase, and then transformed into competent E. coli cells (DH5α). Positive clones were selected on LB agar plates containing the appropriate antibiotic (100 µg/mL ampicillin).

The plasmid DNA from selected clones was extracted via a miniprep kit and subjected to verification by restriction enzyme mapping to confirm the correct insert size and Sanger sequencing, using vector-specific primers (CMV forward and BGH reverse primers) to ensure 100% sequence accuracy of the insert and its reading frame. Colony PCR was performed using a forward primer annealing to the vector backbone and a reverse primer specific to the inserted *F* gene sequence, yielding an amplicon of 427 bp for positive clones.

#### 2.3.4. Master Seed Stock Preparation

A single, sequence-verified positive colony was inoculated into a starter culture, which was then expanded to prepare a large-volume Master Cell Bank (MCB) of the recombinant bacterial strain. Plasmid DNA from the MCB was prepared on a midi-scale, aliquoted, and stored at −80 °C as the Master Plasmid Stock for all subsequent large-scale productions.

#### 2.3.5. In Vitro Expression Validation of the DNA Vaccine Construct

##### Cell Culture

HEK-293T (human embryonic kidney) and CHO-K1 (Chinese hamster ovary) cells were obtained from the Cell Bank of the Chinese Academy of Sciences, Shanghai, China. HEK-293T cells were cultured in Dulbecco’s modified Eagle medium (DMEM; Gibco, Grand Island, NY, USA) supplemented with 10% fetal bovine serum (FBS; Gibco, USA) and 1% penicillin–streptomycin (100 U/mL penicillin, 100 μg/mL streptomycin; Sigma (St. Louis, MO, USA)). CHO-K1 cells were maintained in Ham’s F-12K medium (Corning, Corning, NY, USA) containing 10% FBS and 1% penicillin–streptomycin. Both cell lines were incubated at 37 °C in a humidified atmosphere containing 5% CO_2_. Cells were subcultured every 3–4 days using 0.25% trypsin–EDTA (Gibco, USA) upon reaching 80–90% confluence.

##### Transfection

For transfection, cells were seeded into 24-well plates at a density of 1.0–1.5 × 10^5^ cells per well in 500 μL of antibiotic-free growth medium and cultured overnight to reach 70–80% confluence at the time of transfection. Transfection was performed using Lipofectamine 3000 reagent (Invitrogen, Carlsbad, CA, USA) according to the manufacturer’s instructions. Briefly, for each well, 0.5 μg of plasmid DNA (pCMV-*IL-18*/NDV-*F* or empty vector control) was diluted in 25 μL of Opti-MEM I reduced serum medium (Gibco, USA), and 1 μL of P3000 reagent was added. Separately, 1.5 μL of Lipofectamine 3000 reagent was diluted in 25 μL of Opti-MEM I. The diluted DNA and Lipofectamine 3000 were combined, mixed gently, and incubated for 10–15 min at room temperature to form DNA–lipid complexes. The mixture (50 μL per well) was then added dropwise to the cells. After 6 h of incubation, the medium was replaced with fresh complete growth medium. Cells were harvested 48 h post-transfection for RNA extraction.

##### RNA Extraction

Total RNA was extracted from transfected cells using TRIzol reagent (Invitrogen, USA) according to the manufacturer’s protocol. Briefly, the culture medium was removed, and 1 mL of TRIzol reagent was added directly to each well (for a 24-well plate, 0.5 mL per well may be sufficient; adjust accordingly). Cells were lysed by repeated pipetting, and the lysate was transferred to RNase-free microcentrifuge tubes. After incubation at room temperature for 5 min to permit complete dissociation of nucleoprotein complexes, 0.2 mL of chloroform per 1 mL of TRIzol was added. The tubes were shaken vigorously for 15 s, incubated at room temperature for 2–3 min, and centrifuged at 12,000× *g* for 15 min at 4 °C. The aqueous phase was transferred to a new tube, and RNA was precipitated by adding 0.5 mL of isopropanol per 1 mL of TRIzol. After incubation at room temperature for 10 min, samples were centrifuged at 12,000× *g* for 10 min at 4 °C. The RNA pellet was washed with 1 mL of 75% ethanol, centrifuged at 7500× *g* for 5 min at 4 °C, air-dried for 5–10 min, and dissolved in RNase-free water. RNA concentration and purity were assessed using a NanoDrop spectrophotometer (Thermo Fisher Scientific, Wilmington, DE, USA) by measuring absorbance at 260 nm and 280 nm (A260/A280 ratio between 1.8 and 2.0 was considered acceptable).

##### Reverse Transcription and Quantitative Real-Time PCR (qPCR)

First-strand cDNA was synthesized from 1 μg of total RNA using a PrimeScript RT reagent kit with gDNA Eraser (Takara, Kyoto, Japan) following the manufacturer’s instructions. qPCR was performed using TB Green Premix Ex Taq II (Takara, Japan) on a QuantStudio 5 (Applied Biosystems, Foster City, CA, USA) real-time PCR system. The primer sequences were as follows: NDV *F* gene forward: 5′-tgagctgcatctgctcgacg, reverse: 5′-gcggatggaatcacccgaggg; chicken *IL-18* gene forward: 5′-tgagctggaatgcgatgcct, reverse: 5′-ctgaaggcggcggtggttttg; GAPDH (internal control) forward: 5′-ctgcaccaccaa ctgcttag, reverse: 5′-gtctgggatggaaattgtga. Each reaction (20 μL) contained 10 μL of TB Green Premix, 0.4 μL each of forward and reverse primers (10 μM), 2 μL of cDNA template, and 7.2 μL of RNase-free water. Thermal cycling conditions were initial denaturation at 95 °C for 30 s, followed by 40 cycles of 95 °C for 5 s and 60 °C for 30 s. A melting curve analysis was performed to verify primer specificity. All reactions were performed in triplicate. Relative gene expression levels were calculated using the 2^−ΔΔCt^ method [11], with GAPDH as the reference gene and empty vector-transfected cells as the calibrator (for the NDV *F* gene, undetermined Ct values in control samples were assigned a Ct of 40 as the detection limit for calculation purposes). Data are presented as mean ± SD from three independent experiments. Due to the log-normal distribution of qPCR data, geometric means were used to summarize fold changes, and statistical analyses were performed on log_2_-transformed data. Data are presented as geometric mean ± SD on the log_2_ scale, with fold changes back-transformed for reporting in the text.

### 2.4. Preparation of Inactivated Newcastle Disease Virus Vaccine

#### 2.4.1. Virus Strain and Embryonated Egg Propagation

The vaccine was developed based on the virulent NDV/pigeon/Wenzhou/2023 strain (genotype VII), isolated from pigeons in Wenzhou, Zhejiang Province, China, in 2023. The virus was propagated in specific pathogen-free (SPF) chicken embryos (n = 30). Each embryo was inoculated via the allantoic cavity with 0.1 mL of viral inoculum containing 10^3.5^ 50% egg infectious dose (EID_50_) in sterile saline (0.9% NaCl), supplemented with a 1% antibiotic–antimycotic mixture (benzylpenicillin potassium, kanamycin sulfate, and streptomycin sulfate). Inoculated embryos were incubated at 37.0 °C for 96 h.

#### 2.4.2. Virus Harvest and Titration

Post-incubation, allantoic fluid was harvested and clarified by centrifugation at 3000× *g* for 15 min at 4 °C. The viral titer was determined by performing serial 10-fold dilutions of the harvested fluid in SPF eggs (n = 5 per dilution), followed by incubation at 37.0 °C for 96 h. Hemagglutination (HA) activity of each dilution was assessed, and the final viral titer was calculated to be 10^9.5^ EID_50_/0.1 mL using the Reed and Muench method.

#### 2.4.3. Chemical Inactivation and Validation

Clarified virus harvest was inactivated using a sequential chemical treatment. First, thiomersal was added to a final concentration of 0.01% (*v*/*v*) and incubated at 37 °C for 24 h for its antimicrobial effect. Subsequently, β-propiolactone (BPL) was added to a final concentration of 0.6% (*v*/*v*) and incubated at 37 °C for an additional 24 h to achieve complete nucleic acid inactivation. Following the 24 h inactivation period, the pH of the inactivated virus suspension was measured and adjusted to 7.3–7.4 using sterile 7% NaHCO_3_ solution. The inactivated antigen was then stored at 4 °C for at least 48 h prior to emulsion formulation. No separate neutralization step was required, as BPL undergoes spontaneous and complete hydrolysis under these conditions: at 37 °C, BPL has a half-life of approximately 3.75 h and is completely hydrolyzed within 2–3 h into non-toxic β-hydroxypropionic acid [12]. The prolonged 24 h incubation at 37 °C followed by ≥48 h cold storage ensures that no residual BPL remains in the final antigen preparation. The completeness of inactivation was rigorously confirmed by three consecutive blind passages in SPF eggs (n = 5 per passage). Each passage involved inoculating eggs with 0.1 mL of the treated material (equivalent to an original titer of 10^3.5^ EID_50_/0.1 mL). After 96 h of incubation, allantoic fluid from the third passage was tested for HA activity. The absence of HA activity in all samples confirmed complete viral inactivation.

#### 2.4.4. Emulsion Vaccine Formulation

The inactivated antigen was emulsified with the oil adjuvant Montanide™ ISA 78 VG (SEPPIC S.A., Paris, France) to formulate a water-in-oil (W/O) vaccine. The emulsion was prepared at a ratio of 30:70 (antigen–adjuvant *v*/*v*) using a two-stage homogenization process with an IKA T25 ULTRA-TURRAX homogenizer (IKA®-Werke GmbH & Co. KG, Staufen, Germany): a primary emulsion was formed at 1100 rpm, followed by high-shear homogenization at 4000 rpm to create a stable final emulsion.

#### 2.4.5. Quality Control and Safety Testing

The final vaccine formulation underwent standard quality control assays:

Sterility Test: Samples were inoculated into thioglycollate broth, soybean-casein digest broth, and Sabouraud dextrose broth. No microbial growth was observed after incubation at 37.0 ± 1.0 °C (bacterial media) or 22.5 ± 2.5 °C (fungal medium) for 7 days.

Safety testing was conducted in chickens according to standard pharmacopoeial requirements for inactivated poultry vaccines [13]. Chickens were chosen for safety evaluation because they are the standard model specified by the European Pharmacopoeia (Ph. Eur.) and the Chinese Veterinary Pharmacopoeia for NDV vaccine safety testing, providing a validated and reproducible platform for regulatory compliance [14]. The use of a standardized model ensures that safety data are interpretable within the established regulatory framework. Immunogenicity was subsequently evaluated in the target species (pigeons) as required for efficacy assessment, since immune responses can vary between species, and direct evaluation in the target population is essential for meaningful efficacy conclusions. This two-species approach—safety in a standard model followed by efficacy in the target species—is consistent with established vaccine development pathways. Ten 21-day-old Loman White chickens were administered a 10-fold vaccine dose (0.5 mL) via intramuscular injection into the pectoral muscle. All birds remained clinically healthy throughout the 14-day observation period, with no local reactions (e.g., swelling, necrosis) at the injection site.

**Figure 1:**
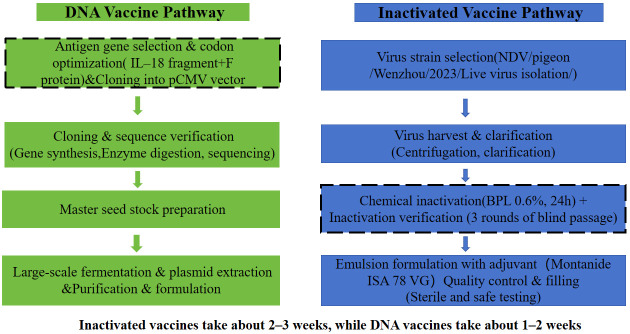
**Workflow comparison of inactivated vaccine and DNA vaccine preparation for pigeon NDV.** Schematic overview of the key steps involved in the production of the DNA vaccine (**left**) and inactivated NDV vaccine (**right**). Arrows indicate sequential stages; dotted boxes highlight critical process differences.

The side-by-side preparation workflows for the inactivated and DNA vaccine platforms are schematically summarized in Figure 1, highlighting the key procedural differences from antigen source to final product.

### 2.5. Experimental Animals and Immunization Protocol

Forty conventional clean-grade Loman White pigeons (a common commercial breed), aged 4 weeks (body weight range: 350–450 g) and of mixed sex (20 males, 20 females), were obtained from a local breeding farm (Wenzhou Pigeon Breeding Cooperative, Zhejiang, China) and used in this study. The birds were conventionally raised with no vaccination history and tested negative for NDV antibodies prior to the experiment, corresponding to a clean-grade health status as defined by Chinese laboratory animal standards. Then, birds were randomly assigned to four groups (n = 10 per group: 5 males, 5 females) using a random number table. (1) PBS control group, (2) NDV DNA vaccine group (pCMV-*IL-18*/NDV-*F*), (3) inactivated vaccine group, and (4) prime–boost combination group (DNA vaccine prime followed by inactivated vaccine boost). All pigeons were housed in separate isolators with ad libitum access to feed and water and were acclimatized for 7 days prior to the experiment.

For immunization, pigeons in the DNA vaccine group received 100 μg of plasmid DNA via intramuscular injection into the pectoral muscle (primary immunization), followed by a booster immunization with the same dose at 14 days post-primary immunization. Birds in the inactivated vaccine group were administered 0.3 mL of the inactivated vaccine via the same intramuscular route. The combination group received the DNA vaccine prime (100 μg) followed by the inactivated vaccine boost (0.3 mL) at 14 days post-primary immunization. The PBS control group was injected with an equivalent volume of phosphate-buffered saline.

Clinical monitoring was performed daily throughout the study period. Parameters assessed included general behavior (alertness, feeding, drinking), local reactions at the injection site (swelling, redness, necrosis), and body weight (measured at days 0, 14, and 28). No adverse reactions were observed in any group. Blood samples were collected from the brachial vein of all birds at baseline (day 0, pre-immunization) and at 14 days after the booster immunization (28 days post-primary immunization). Serum was separated and stored at −20 °C until analysis, and then analyzed for NDV-specific antibodies using the hemagglutination inhibition (HI) assay to evaluate the humoral immune response. The selection of day 14 for booster immunization is supported by both immunological rationale and common practice in avian vaccine research. This interval effectively strikes a balance between allowing the primary immune response to mature and ensuring timely activation of memory immunity, thereby optimizing the synergistic effect. To minimize bias, all HI assays were performed by a technician blinded to group allocation.

### 2.6. Hemagglutination Inhibition (HI) Assay

Serum samples were heat-inactivated at 56 °C for 30 min prior to analysis. The HI assay was performed according to standard protocols using 1% (*v*/*v*) chicken red blood cells (cRBCs) in a V-bottom 96-well microtiter plate. Briefly, two-fold serial dilutions of the inactivated serum were prepared in phosphate-buffered saline (PBS). To each dilution, an equal volume of Newcastle disease virus antigen, containing 4 hemagglutination units (HAU) of the NDV LaSota strain, was added and incubated at room temperature for 30 min. Subsequently, 50 μL of the 1% cRBC suspension was added to each well. The plate was gently mixed and incubated at room temperature for an additional 30–40 min until clear buttons formed in the negative control wells. The HI titer was recorded as the reciprocal of the highest serum dilution that completely inhibited hemagglutination.

## 3. Results

### 3.1. Construction and Verification of the DNA Vaccine Plasmid

The recombinant plasmid encoding the NDV F protein and Gallus IL-18 fragment was successfully constructed (pCMV-*IL-18*/NDV-*F*). Verification by colony-direct PCR using vector-specific primers amplified a fragment of approximately 430 bp, consistent with the expected insert size of 427 bp for the junction fragment, confirming successful ligation of the F-IL-18 fusion gene into the pCMV vector (Supplementary Figure S2). Sequencing confirmed 100% sequence identity and the correct reading frame of the insert. This plasmid was used for all subsequent immunizations. The purified plasmid DNA was obtained at a concentration of 1.081 μg/μL with an A260/A280 ratio of 1.91, indicating high purity.

### 3.2. Inactivation Validation of the NDV Strain

The virulent NDV strain (NDV/pigeon/Wenzhou/2023) was propagated in SPF chicken embryos and inactivated with 0.6% β-propiolactone (BPL). To assess antigenicity retention after inactivation, the hemagglutination (HA) titer of the inactivated viral suspension was measured directly. The HA titer remained at 9 log_2_ (512 HAU/50 μL) following BPL inactivation and pH adjustment, identical to the pre-inactivation titer. This indicates that the inactivation process did not compromise viral antigenicity.

To confirm complete inactivation, the treated material was subjected to three consecutive blind passages in SPF eggs. Allantoic fluid harvested from the third passage was tested for HA activity. No HA activity was detected in any sample, confirming the absence of replicating virus and validating the safety of the inactivated antigen preparation.

### 3.3. Immunogenicity of the Vaccine Candidates

The immunogenicity of the DNA vaccine, inactivated vaccine, and their combination was evaluated in pigeons (n = 10) by measuring hemagglutination inhibition (HI) antibody titers against NDV. All statistical analyses were performed on log_2_-transformed HI titers using GraphPad Prism (version 9.0, GraphPad Software, San Diego, CA, USA). Normality of data distribution was confirmed using the Shapiro–Wilk test (*p* > 0.05 for all groups), and homogeneity of variances was verified by Levene’s test (*p* = 0.32). As shown in Table 1, one-way ANOVA indicated a highly significant main effect of the immunization regimen on antibody titers (*F*(3, 36) = 24.68, *p* < 0.0001). Post hoc Tukey’s test revealed that all vaccinated groups elicited significantly higher antibody responses compared to the unvaccinated control group (1.78 ± 0.67) (all *p* < 0.0001). While no significant difference in titer was observed between the DNA vaccine group (8.00 ± 0.71) and the inactivated vaccine group (7.00 ± 0.82) (*p* = 0.25; mean difference = 1.00, 95% CI: −0.52 to 2.52), the prime–boost combination group (10.63 ± 0.52), which received DNA prime followed by inactivated vaccine boost, generated significantly higher antibody titers than either the DNA vaccine alone (*p* = 0.003; mean difference = 2.63, 95% CI: 1.11 to 4.15) or the inactivated vaccine alone (*p* = 0.0005; mean difference = 3.63, 95% CI: 2.11 to 5.15).

Body weights were measured at days 0, 14, and 28 post-primary immunization. No significant differences in body weight were observed between any of the vaccinated groups and the control group at any time point. All birds maintained normal weight gain throughout the study period, consistent with the absence of adverse clinical signs reported in Section 2.5.

### 3.4. Expression Validation of the DNA Vaccine Construct

To verify the transcriptional activity of the DNA vaccine construct, qPCR was performed on transfected HEK-293T and CHO-K1 cells. In cells transfected with the empty vector (negative control), NDV *F* gene amplification remained below the detection limit (Ct > 40). Conversely, cells transfected with pCMV-*IL-18*/NDV-*F* exhibited robust *F* gene expression. In HEK-293T cells, ΔCt values (*F* gene − GAPDH) ranged from 5.24 to 13.81 (Supplementary Table S1; Figure S4A), corresponding to a geometric mean upregulation of 393-fold (range: 15- to 5740-fold; corresponding to log_2_-transformed mean ± SD of 8.62 ± 3.93) after normalization (Figure S4B). A similar expression pattern was observed in CHO-K1 cells, with a geometric mean increase of 297-fold (range: 36- to 679-fold; log_2_-transformed mean ± SD: 8.16 ± 0.86). Chicken *IL-18* transcripts were detected in both control and transfected cells (ΔCt range: 0.81–1.65; Figure S4C), with a modest upregulation (approximately 2.5-fold) observed upon transfection (Figure S4D).

## 4. Discussion

This study demonstrates that a heterologous prime–boost regimen, using a DNA vaccine followed by an inactivated vaccine, elicits a significantly stronger humoral immune response against pigeon NDV than either vaccine platform alone. This synergistic effect provides a compelling rationale for integrating these complementary technologies.

### 4.1. Vaccine Design and Rationale

The DNA vaccine was designed to encode the fusion (F) protein of NDV, a major protective antigen critical for viral entry and a key target for neutralizing antibodies [15]. To enhance cell-mediated immunity, we employed a molecular adjuvant strategy by constructing a fusion gene incorporating the F protein with chicken interleukin-18 (IL-18). Although the pigeon *IL-18* sequence was unavailable, the chicken homolog was selected based on the high conservation and demonstrated cross-reactive bioactivity of cytokines among avian species [16,17,18]. IL-18 is a well-characterized adjuvant that promotes Th1-type responses and enhances cytotoxic T lymphocyte activity [19,20,21]. The fusion format ensures co-delivery and co-expression of the antigen and adjuvant within the same target cells, potentially optimizing immunostimulation [22]. The gene cassette was cloned into the pCMV vector, utilizing its strong CMV promoter to drive high-level expression in vivo [3].

### 4.2. Mechanistic Hypothesis for the Observed Synergy

The significantly higher HI antibody titers elicited by the DNA prime-inactivated boost regimen suggest a genuine synergistic interaction rather than a simple additive effect. Understanding the mechanism underlying this synergy is critical for optimizing the strategy. While direct cellular evidence is lacking, a plausible hypothesis can be proposed based on the classical principle of heterologous prime–boost immunization.

We hypothesize that the DNA prime may establish a broad foundation of immune recognition, potentially by inducing antigen-specific T helper cells (Th) and cytotoxic T lymphocytes (CTLs)—an effect that could be facilitated by the co-expressed chicken IL-18 adjuvant. This cytokine is known to promote a Th1-biased response, which is crucial for cellular immunity and for providing optimal help to B cells. Subsequently, the boost with the inactivated vaccine—which presents a high density of conformationally intact viral antigens in a repetitive array (especially when formulated with an oil adjuvant)—may drive B cell clonal expansion and differentiation, preferentially stimulating the memory B cells and T helper cells generated by the DNA prime. This proposed sequence of events, however, remains inferred from the observed HI antibody data and the established literature on IL-18 and vaccine platforms, rather than being directly demonstrated by cellular immune assays in this study.

The use of chicken IL-18 in pigeons is justified by the high conservation of cytokine sequences and reported cross-species bioactivity among avian species [9,10,12]; nevertheless, its precise immunological effects in pigeons—particularly the extent to which it promotes Th1/CTL responses—remain to be empirically confirmed. Future studies incorporating direct measurements of cell-mediated immunity, such as IFN-γ ELISpot assays or flow cytometric analysis of T-cell subsets, are essential to validate the proposed mechanistic pathway.

### 4.3. Strategic Application: A Dual-Platform Sequential Immunization Framework

**Figure 2:**
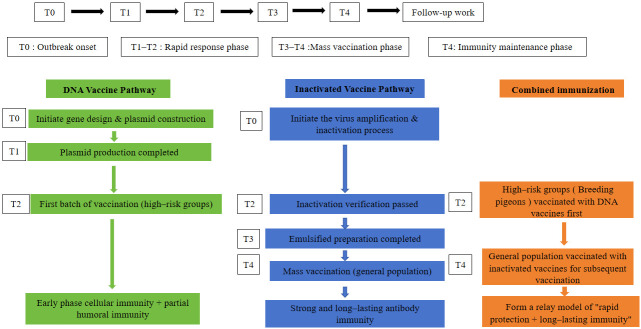
**Proposed synergistic immunization strategy combining DNA vaccine and inactivated vaccine platforms for rapid response to NDV outbreaks.** Timeline-based strategy illustrating parallel development and sequential deployment of DNA vaccine (for rapid early protection) and inactivated vaccine (for broad and durable immunity) in an outbreak scenario.

Building on this observed synergy, we propose a practical dual-platform sequential immunization strategy for outbreak management (Figure 2). This strategy capitalizes on the distinct strengths of each platform: the rapid design and production capabilities of DNA vaccines for early containment and the ability of inactivated vaccines to provide durable, high-titer protection. During the initial outbreak phase (T0), the DNA vaccine platform could be deployed within weeks to vaccinate high-risk populations (e.g., priority pigeon flocks), offering early protection and inducing foundational cellular immunity. Concurrently, scaled production of an inactivated vaccine based on the local epidemic strain would be initiated. By the T4 stage (4–8 weeks post-prime), when the inactivated vaccine is ready, a booster could be administered to primed individuals, and large-scale vaccination could be extended to the broader population.

**Figure 3:**
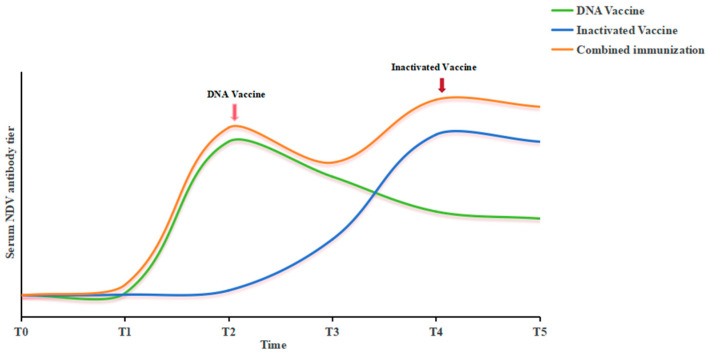
**Collaborative immunization strategy**: parallel development and sequential vaccination of DNA vaccines and inactivated vaccines.

The essence of this framework (Figure 3) is to translate our experimental paradigm into a phased, actionable public health strategy. It effectively integrates the speed and design flexibility of DNA vaccines with the robust immunogenicity and technological maturity of inactivated vaccines (Table 2). This conceptual framework represents a theoretical roadmap for integrating the complementary strengths of both platforms. However, its practical applicability for outbreak response remains to be empirically validated. Key questions that must be addressed in future work include (i) whether the enhanced HI titers observed here correlate with protection against viral challenge, (ii) whether the immune response is sustained beyond 28 days post-immunization, and (iii) whether the sequential regimen is logistically feasible and cost-effective in field settings.

### 4.4. Methodological Considerations and Limitations

Several limitations of this study should be acknowledged:

#### 4.4.1. Sample Size and Statistical Power

The sample size (n = 10 per group) aligns with common practice in preliminary avian immunogenicity trials. While a larger cohort would provide greater statistical power to detect subtle differences, the highly significant effects observed (*p* < 0.0001 between vaccinated groups and control and a clear synergy in the prime–boost group) indicate that the primary outcomes were robustly detectable with the current design. Importantly, the lack of a statistically significant difference between the DNA-only and inactivated-only groups (*p* = 0.25) should be interpreted with caution; this may reflect insufficient power to detect a modest difference rather than true equivalence.

#### 4.4.2. Absence of Challenge Data

While the significantly elevated HI antibody titers in the prime–boost group are suggestive of enhanced protective potential—given the well-established correlation between HI titers and protection against NDV—immunogenicity does not directly equate to protective efficacy. The absence of viral challenge data is a limitation, and whether the heightened humoral response translates into reduced morbidity, mortality, or viral shedding remains to be demonstrated.

#### 4.4.3. Single Time Point and Lack of Cellular Data

Antibody responses were assessed at a single time point (28 days post-prime), capturing peak responses but providing no information on immune kinetics or durability. Additionally, cellular immune responses were not measured; the proposed mechanism involving IL-18-mediated Th1 bias, therefore, remains hypothetical and requires direct validation.

#### 4.4.4. Heterologous HI Antigen

The HI assay used LaSota antigen (genotype II) to detect antibodies induced by genotype VII-based vaccines. While LaSota is the internationally recognized reference strain for NDV serology [23], and cross-reactivity within the serotype is well documented, previous studies have shown that homologous antigen yields approximately 2-fold higher titers [24]. Therefore, the absolute HI values reported here may underestimate the true antibody levels. However, because all groups were tested with the same standardized antigen, the relative differences between groups remain valid.

#### 4.4.5. In Vitro Expression Validation

qPCR confirmed robust transcription of the NDV *F* gene from our DNA vaccine construct, but IL-18 transcript upregulation was modest, possibly due to high background signal in control cells. Whether the fusion protein is efficiently translated and whether the IL-18 moiety retains biological activity in pigeons requires further investigation through protein-level analysis (e.g., Western blot) or functional assays.

While the use of HEK-293T and CHO-K1 cells confirms transcriptional activity of the DNA vaccine construct, these were mammalian cell lines and may not fully recapitulate expression in avian cells. The DF-1 chicken fibroblast cell line was a well-established model for avian gene expression studies [25] and would provide more translationally relevant data. Future studies should include validation in avian cell lines such as DF-1 or primary chicken embryo fibroblasts (CEFs) to confirm that the construct is efficiently expressed in cells of the target species.

#### 4.4.6. Clinical Monitoring and Species Discontinuity

Although clinical monitoring was performed, systematic scoring of behavioral changes and injection site reactions was not conducted. The use of chickens for safety testing and pigeons for immunogenicity, while justified by regulatory standards, introduces a species discontinuity; however, the absence of adverse effects in chickens supports the vaccine’s safety profile.

### 4.5. Practical Considerations for Field Implementation

Beyond immunological synergy, the practical implementation of a dual-platform sequential immunization strategy in field settings warrants consideration. Beyond immunogenicity, practical implementation requires consideration of cold chain logistics: inactivated vaccines typically require refrigerated storage (2–8 °C), whereas DNA vaccines exhibit greater thermostability and could potentially be stored as lyophilized preparations at ambient temperatures, offering logistical advantages in resource-limited settings [26]. Administration logistics also present distinct challenges: inactivated vaccines are supplied in ready-to-use liquid formulations, while DNA vaccines may require delivery optimization (e.g., electroporation, lipid nanoparticles) to achieve optimal immunogenicity [27]. Cost implications are multifaceted: DNA vaccines offer rapid production and flexible antigen updating but may have higher upfront development costs, whereas inactivated vaccines benefit from established manufacturing infrastructure but involve live virus handling and downstream processing expenses [28]. These factors must be carefully balanced when translating laboratory findings into practical vaccination programs.

### 4.6. Future Directions

Future confirmatory studies with larger animal cohorts are warranted to validate the observed synergy, investigate cellular immune parameters, and assess protective efficacy in challenge models. Studies incorporating multiple sampling time points (e.g., weekly intervals) and long-term follow-up (e.g., 3–6 months) would characterize immune kinetics and durability. The use of genotype-matched antigens would provide more accurate absolute titer values for genotype VII-specific immunity. Additionally, protein-level validation of IL-18 expression and bioactivity would clarify the adjuvant’s immunostimulatory capacity. Finally, field trials assessing logistical feasibility, cost-effectiveness, and protective efficacy under real-world conditions will be essential to translate this experimental strategy into practical application. Additionally, protein-level validation of IL-18 expression and bioactivity in avian cell lines (e.g., DF-1) is needed to confirm that the fusion protein is efficiently translated and that the IL-18 moiety retains immunostimulatory activity in the target species.

## 5. Conclusions

In summary, this study demonstrates that a heterologous DNA prime-inactivated boost regimen elicits significantly higher HI antibody titers in pigeons than either vaccine alone, indicating a clear synergistic effect on humoral immunogenicity. These findings highlight the complementary potential of the two platforms and provide a strong immunological rationale for further development of this sequential strategy. Future studies incorporating viral challenge experiments and long-term immune monitoring are needed to determine whether this enhanced antibody response translates into protective efficacy and durable immunity under field conditions.

## Figures and Tables

**Table 1:** Hemagglutination inhibition (HI) antibody responses in pigeons immunized with different vaccine regimens against Newcastle disease (28 days post-primary immunization).

Immunization Group	HI Antibody Titer (log_2_, Mean ± SD)
Unvaccinated Control	1.78 ± 0.67 ^a^
DNA Vaccine	8.00 ± 0.71 ^b^
Inactivated Vaccine	7.00 ± 0.82 ^b^
DNA + Inactivated Vaccine	10.63 ± 0.52 ^c^

Different superscript letters (a, b, c) indicate statistically significant differences at *p* < 0.05 (one-way ANOVA followed by Tukey’s post hoc test). Means sharing the same superscript letter are not significantly different.

**Table 2:** Comparative characteristics of inactivated vaccine and DNA vaccine platforms for NDV.

Feature Dimension	Inactivated Vaccine	DNA Vaccine	Platform Positioning & Recommendation
Development Timeline	Slower (relies on virus cultivation & inactivation, 2–4 weeks)	Faster (gene synthesis & plasmid production, 1–2 weeks)	
Type of Immune Response	Primarily humoral immunity (strong antibody response)	Both humoral and cellular immunity (advantageous for clearing intracellular viruses)	
Platform Positioning	Mass vaccination for long-lasting protection	Rapid response for emergency use	Inactivated vaccine was recommended for large-scale routine immunization or booster strategies that aim to provide durable and high-titer antibody protection. DNA vaccine was best applied for rapid deployment in response to outbreaks caused by novel or variant viruses, as well as in scenarios where the induction of cellular immunity is a key objective, such as in preclinical research.

This comparison integrates established characteristics of each platform with observations from the present study.

## Data Availability

The data presented in this study are available upon request from the corresponding author due to the data being part of an ongoing research project.

## Supplementary Materials

The following supporting information can be downloaded at: https://www.mdpi.com/article/10.3390/vetsci13030251/s1, Supplementary Figure S1: Recombinant plasmid pCMV-*IL-18*/NDV-*F*; Supplementary Figure S2: pCMV-*IL-18*/NDV-*F* verification by colony-direct PCR; Supplementary Figure S3: pCMV-*IL-18*/NDV-*F* sequencing confirmed; Figure S4: In vitro expression validation of pCMV-*IL-18*/NDV-*F* in HEK-293T cells and CHO-K1 cells; Table S1: qPCR data summary for NDV-*F* and chicken *IL-18* expression in transfected HEK-293T and CHO-K1 cells.
