# Supplementary material for: A DNA Prime-Inactivated Boost Regimen Enhances Immunogenicity Against Pigeon Newcastle Disease: A Comparative Study and Analysis of Synergistic Effects
Source: Vet Sci. 2026 Mar 9;13(3):251. doi: 10.3390/vetsci13030251 (PMC13029869; doi:10.3390/vetsci13030251)
Supplement: Supplementary file 1 [file vetsci-13-00251-s001.zip › Supplementary Figure.pdf]

Figure S1

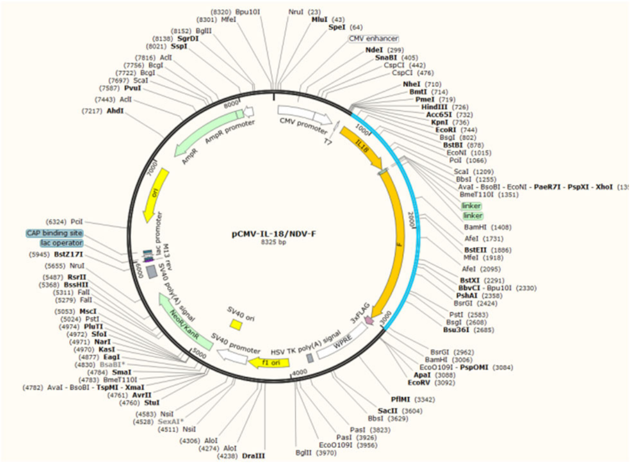

Supplementary Figure S1: Recombinant plasmid pCMV-*IL-18*/NDV-*F*.

Figure S2

A

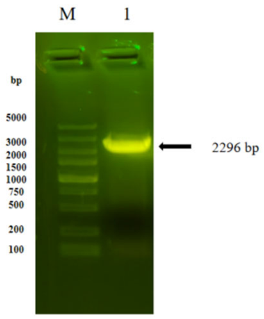

Figure S2A.Gel electrophoresis pattern of PCR amplification product of target gene

M DL 5000

1 IL18+NDV-F

B

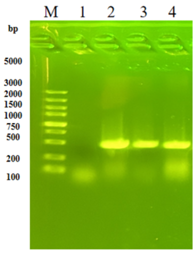

Supplementary Figure S2B. Colony PCR verification of pCMV-IL-18/NDV-F using a vector-specific forward primer and an insert-specific reverse primer. Lanes 2-4: Representative positive colonies showing the expected ~430 bp amplicon, confirming successful ligation of the F-IL-18 fusion gene. Lane M: DNA marker; Lane 1: Negative control (water).

Supplementary Figure S2: pCMV-*IL-18*/NDV-*F* verification by colony-direct PCR. (A)Gel electrophoresis pattern of PCR amplification product of target gene；(B)Colony PCR verification of pCMV-*IL-18*/NDV-*F*.

Figure S3

A

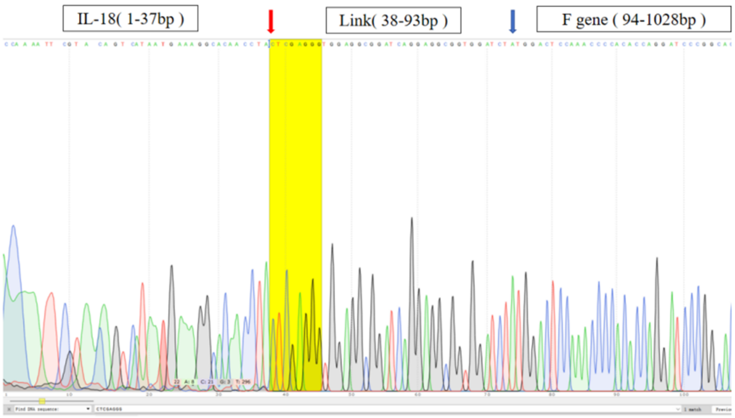

B

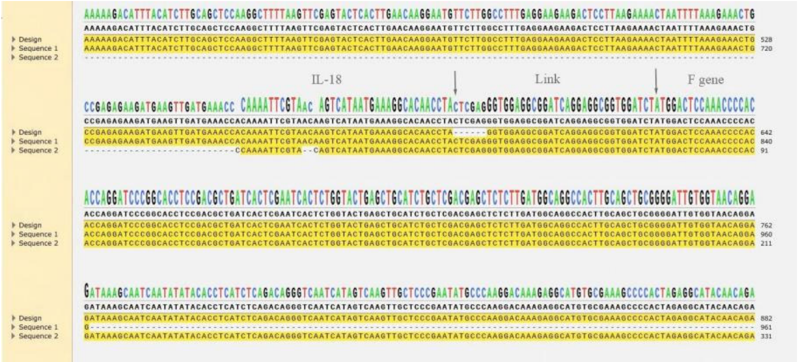

C

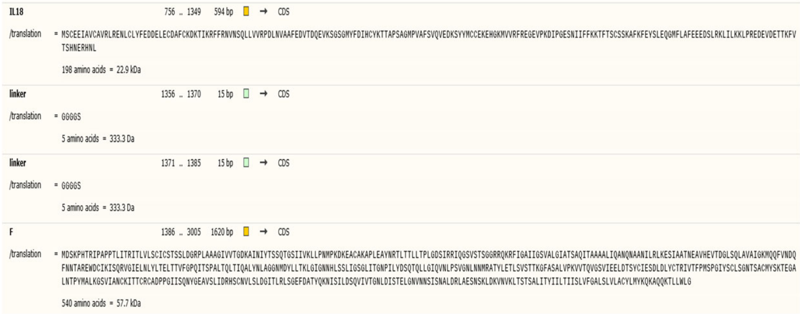

Supplementary Figure S3. Sequence verification of the IL-18-F fusion junction.

(A) Chromatogram of the junction region, showing clean, unambiguous peaks. The vertical dashed line indicates the fusion boundary between the chicken *IL-18* gene (left) and the NDV *F* gene (right).

(B) Nucleotide sequence alignment between the theoretical sequence (top), the sequencing 1 result and sequencing 2 result (bottom). yellow coler indicate 100% identity. The fusion junction is marked by a arrow.

(C) Translated amino acid sequence, demonstrating that the fusion is in-frame. The IL-18 sequence is followed by link and the F protein sequence, confirming correct construction.

Figure S4

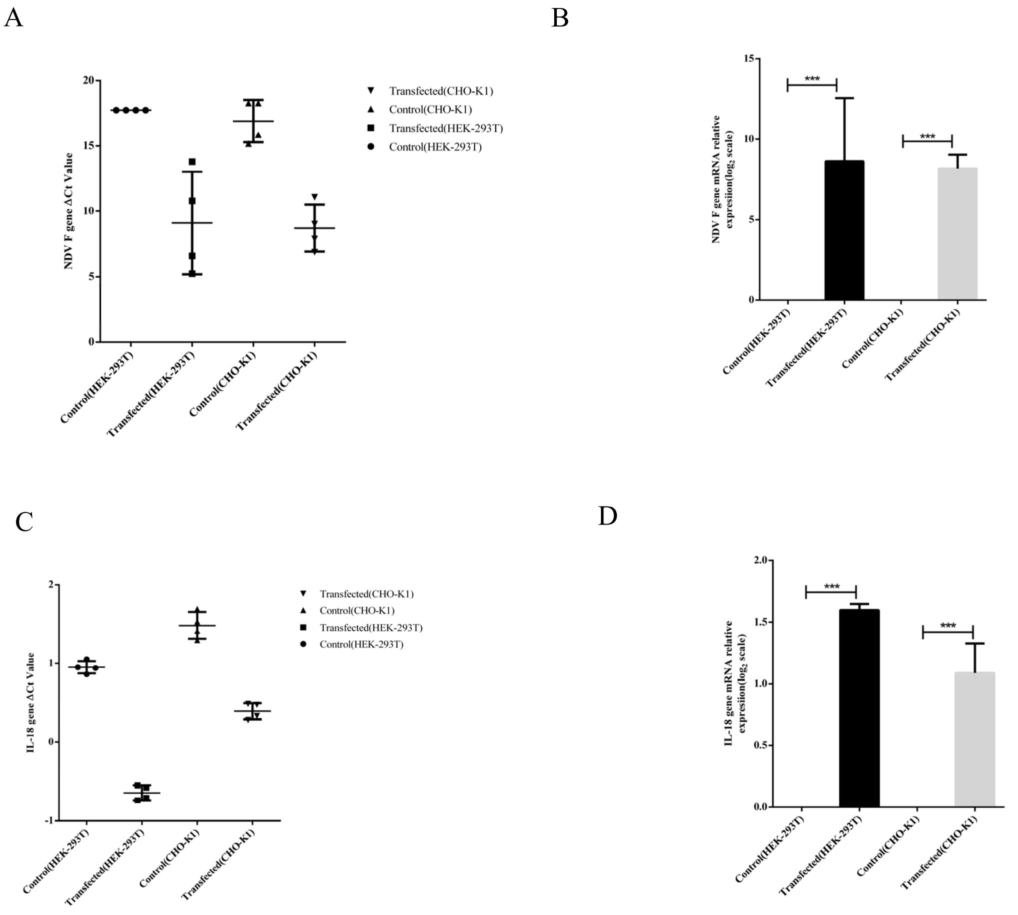

Supplementary Figure S4. In vitro expression validation of pCMV-*IL-18*/NDV-*F* in HEK-293T cells and CHO-K1 cells. Cells were transfected with the DNA vaccine construct or empty vector control, and total RNA was analyzed by qPCR at 48 h post-transfection. (A) NDV *F* gene expression. Individual data points represent ΔCt values (*F* gene – GAPDH); lower ΔCt indicates higher expression. *F* gene amplification was undetectable in controls (Ct > 40). (B) Relative *F* gene expression normalized to GAPDH and calibrated to empty vector controls (Ct = 40 assigned as detection limit). Data are presented as geometric mean ± SD on $\log_2$ scale (back-transformed values: 393-fold; range 15- to 5740-fold) in HEK-293T cells and (back-transformed values: 297-fold; range: 36- to 679-fold) in CHO-K1 cells. (C) Chicken *IL-18* ΔCt values. (D) Relative IL-18 expression showing modest upregulation (approximately 2.5-fold). All data represent three independent experiments with four technical replicates each. ***$p < 0.001$; ns, not significant.
