# Supplementary figures and images for: A DNA Prime-Inactivated Boost Regimen Enhances Immunogenicity Against Pigeon Newcastle Disease: A Comparative Study and Analysis of Synergistic Effects
Source: Vet Sci. 2026 Mar 9;13(3):251. doi: 10.3390/vetsci13030251 (PMC13029869; doi:10.3390/vetsci13030251)

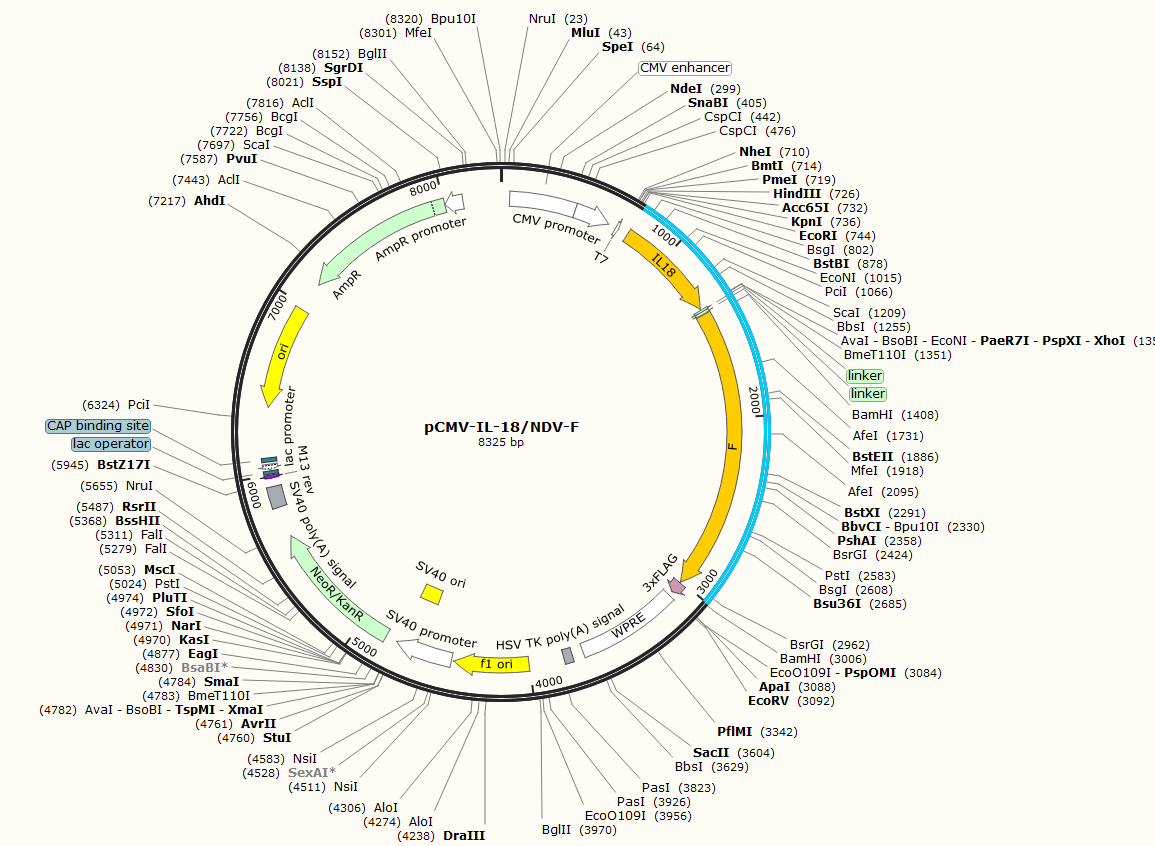

Supplement: Supplementary file 1 [file vetsci-13-00251-s001.zip › Figure S1/Figure S1.jpg]

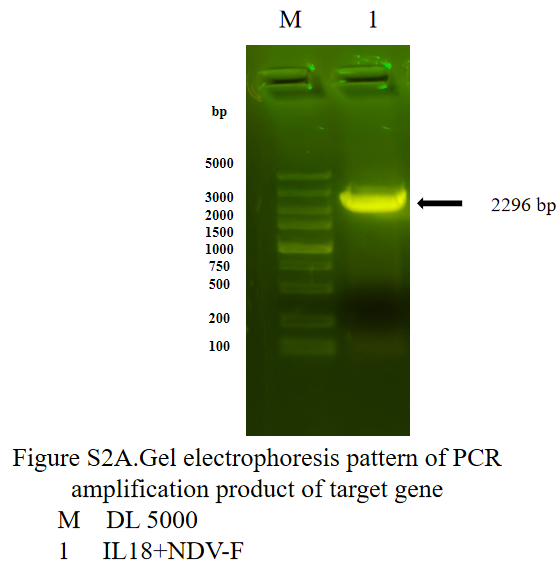

Supplement: Supplementary file 1 [file vetsci-13-00251-s001.zip › Figure S2/Figure S2A.jpg]

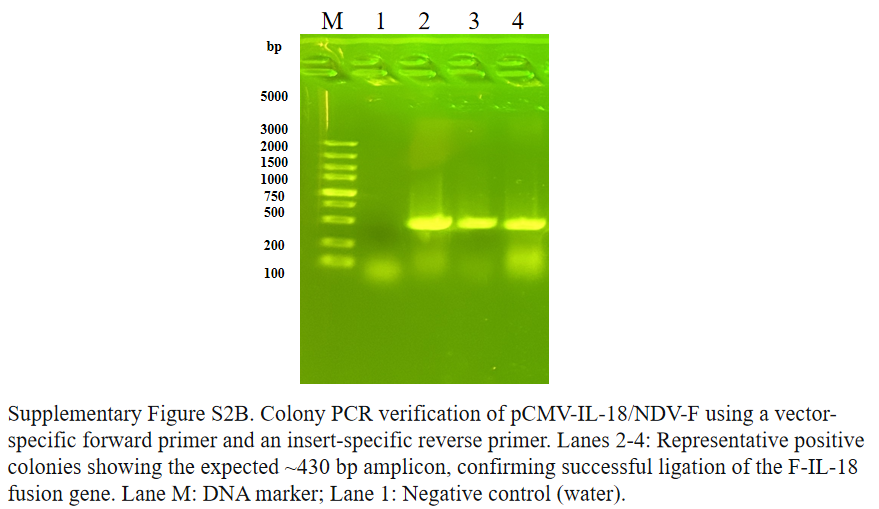

Supplement: Supplementary file 1 [file vetsci-13-00251-s001.zip › Figure S2/Figure S2B.jpg]

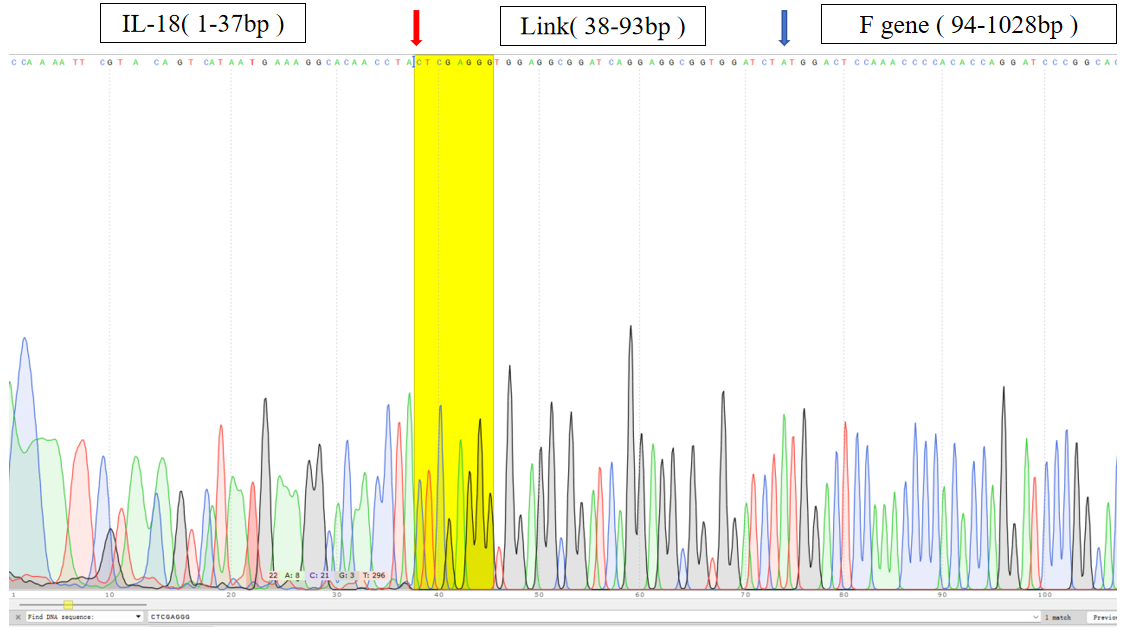

Supplement: Supplementary file 1 [file vetsci-13-00251-s001.zip › Figure S3/Figure S3A.jpg]

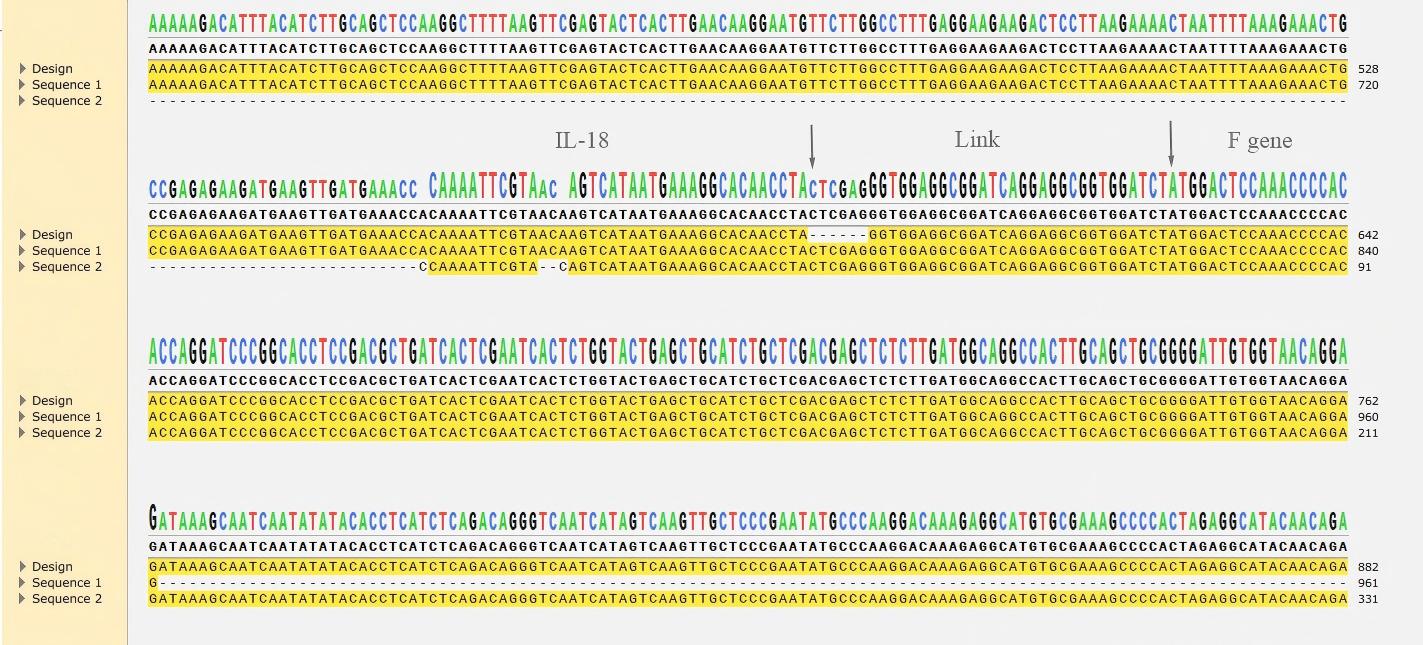

Supplement: Supplementary file 1 [file vetsci-13-00251-s001.zip › Figure S3/Figure S3B.jpg]

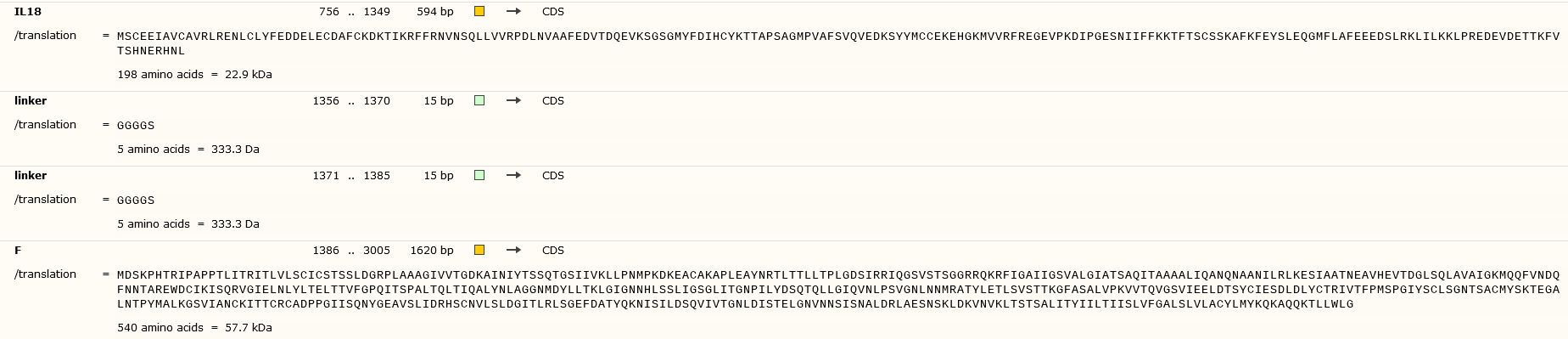

Supplement: Supplementary file 1 [file vetsci-13-00251-s001.zip › Figure S3/Figure S3C.jpg]

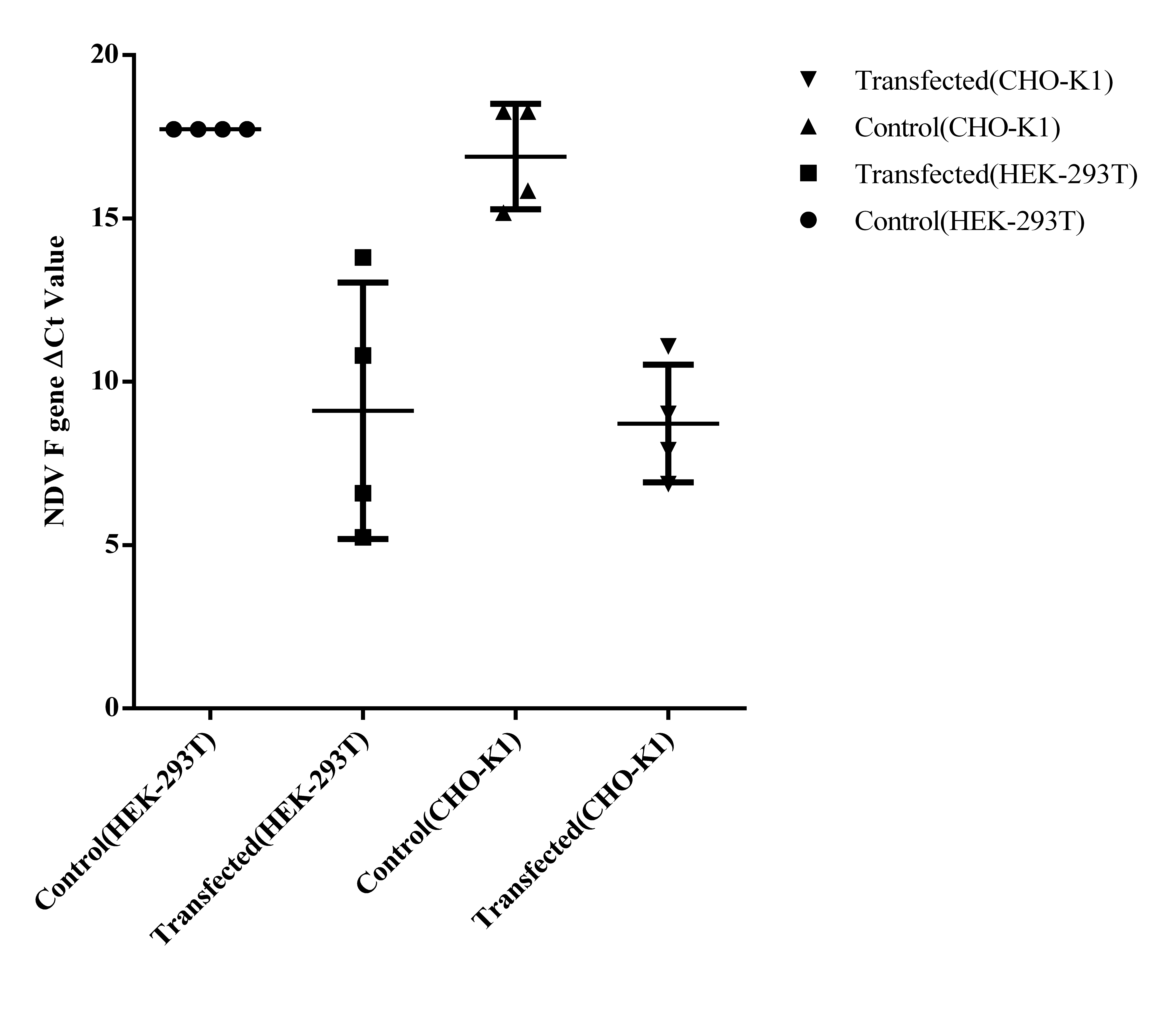

Supplement: Supplementary file 1 [file vetsci-13-00251-s001.zip › Figure S4/Figure S4A.tif]

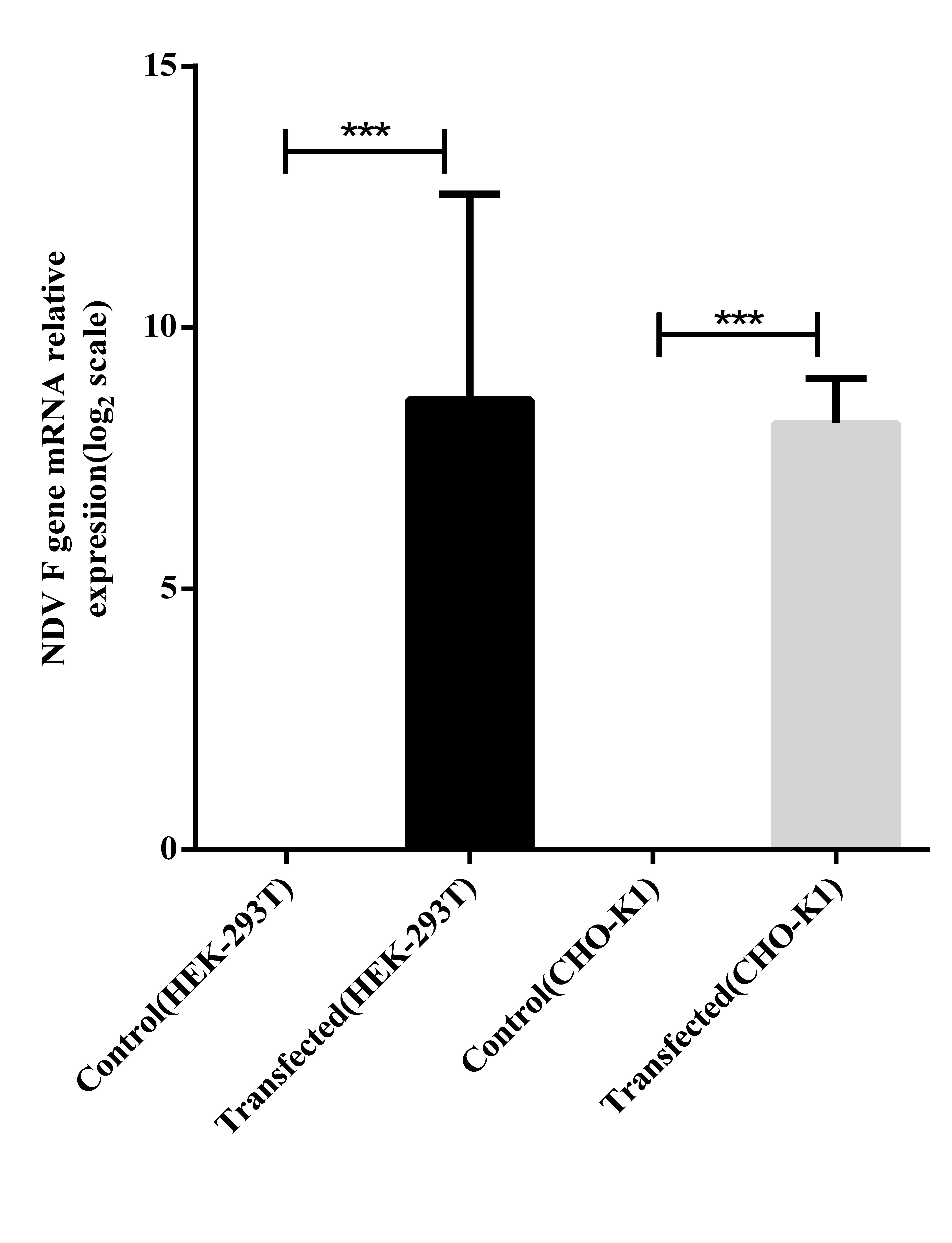

Supplement: Supplementary file 1 [file vetsci-13-00251-s001.zip › Figure S4/Figure S4B.tif]

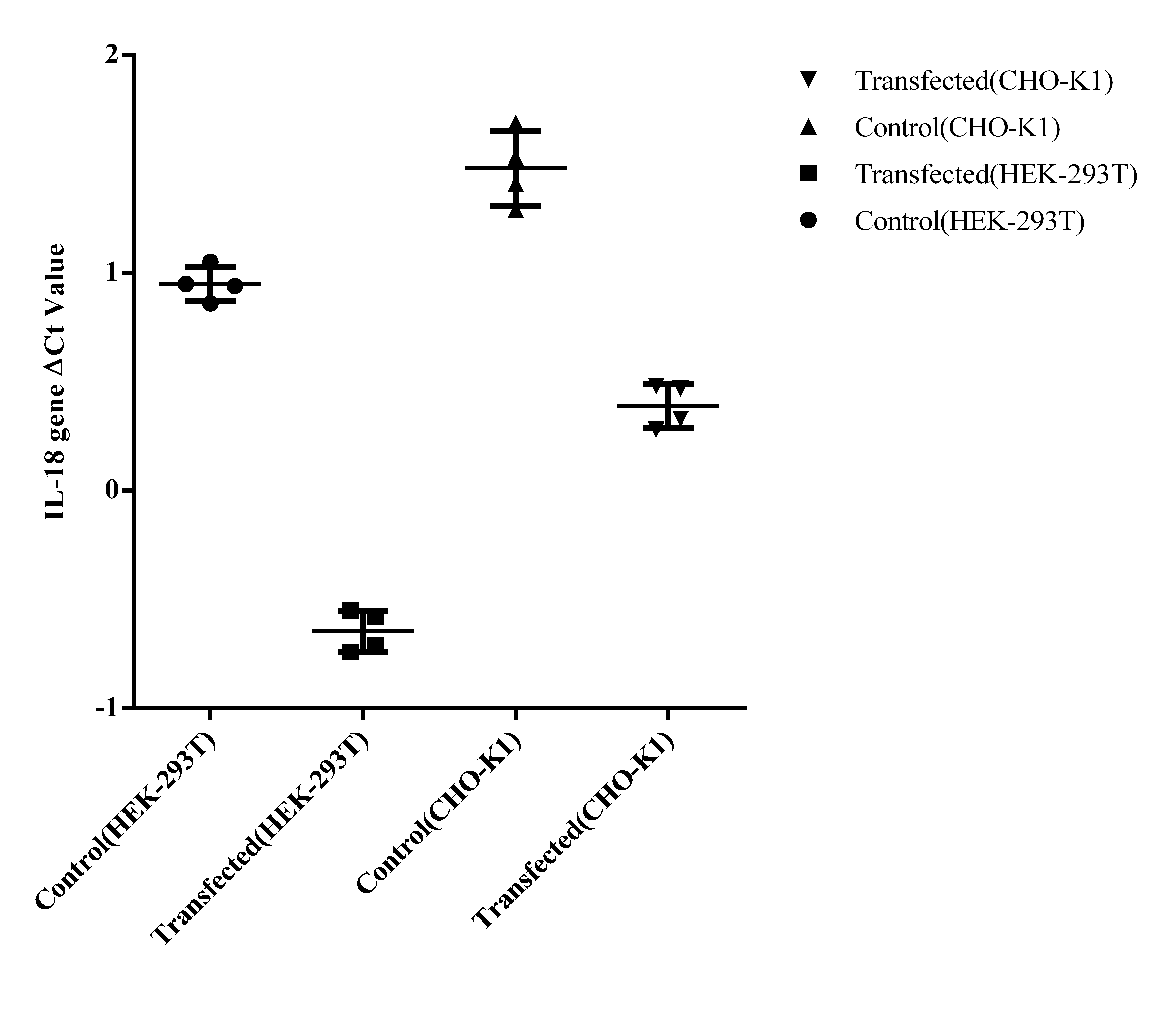

Supplement: Supplementary file 1 [file vetsci-13-00251-s001.zip › Figure S4/Figure S4C.tif]

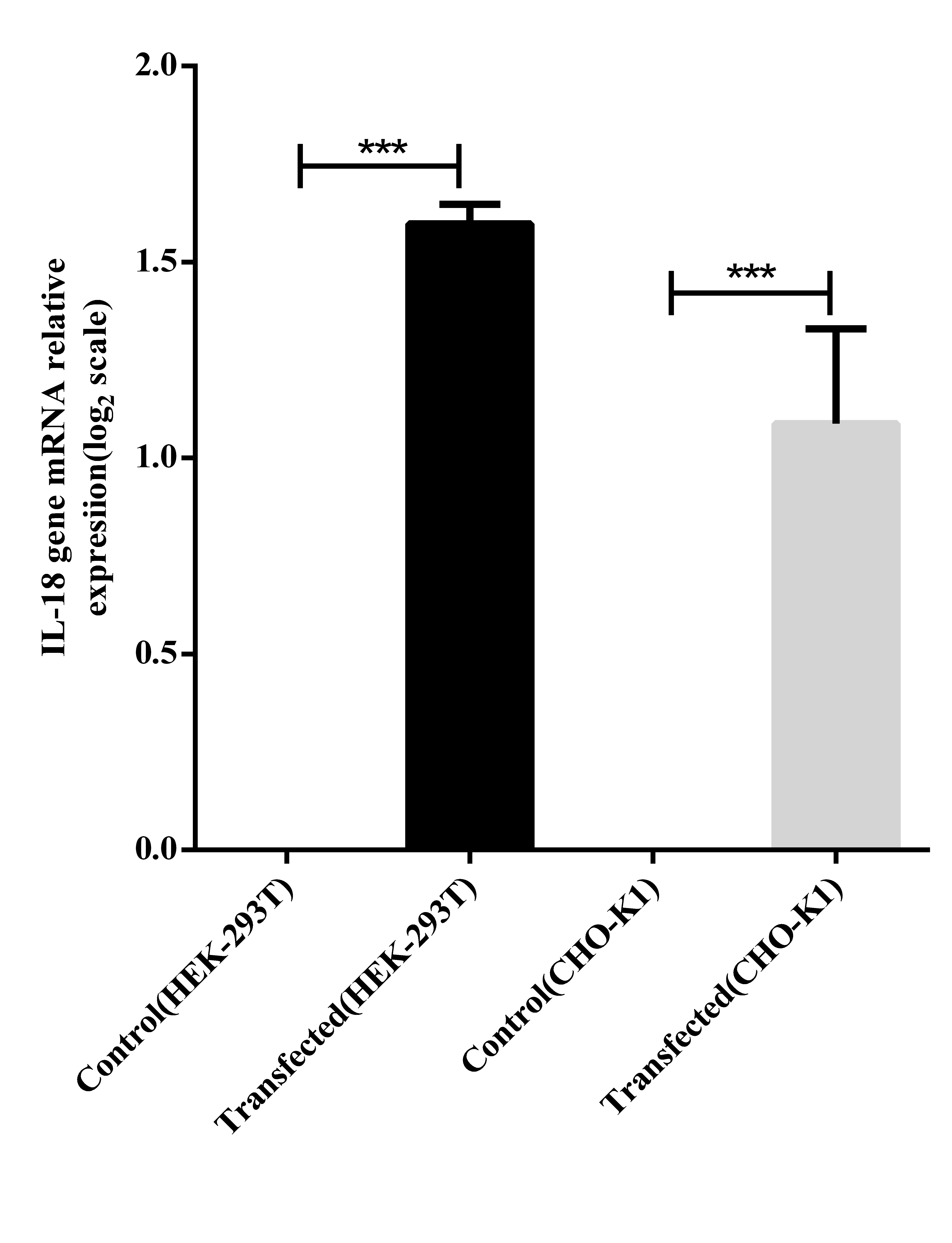

Supplement: Supplementary file 1 [file vetsci-13-00251-s001.zip › Figure S4/Figure S4D.tif]
